# Comparison of Application of Mitomycin C Vs Silicon Stenting Vs Conventional Method in Endonasal Dacrocystorhinostomy: A Randomized Controlled Trial of 150 Patients

**Published:** 2018-01

**Authors:** Shrinivas- Shripatrao Chavan, Vitthal D Kale, Karthik N Rao, Devkumar Rengaraja, Amol Hekare

**Affiliations:** 1 *Department of Otorhinolaryngology, Grant Medical College And Sir Jj Group Of Hospitals, Byculla, Mumbai, India.*

**Keywords:** Conventional, Dacrocystitis, Endonasal dacrocystorhinostomy, Mitomycin C, Silicone tubing

## Abstract

**Introduction:**

Since the days of Hippocrates, many modifications have been proposed in endonasal dacrocystorhinostomy (DCR), with the use of new drugs and implants showing variable results. The objective of this study was to analyze whether the use of silicon tubing or mitomycin C administration has an added advantage over conventional endonasal DCR.

**Materials and Methods:**

A randomized controlled trial of 150 patients between the ages of 6 and 70 years presenting with epiphora was performed. Patients were randomly divided into three groups: endonasal DCR with mitomycin C administration, endonasal DCR with silicon stenting, or conventional endonasal DCR. Patients were followed up on Days 15, 30, 60 and 90 postoperatively for sac syringing to confirm patency.

**Results:**

The majority of patients (28.7%) were in the fourth decade of life, with a female predominance (65.3%). Dacrocystitis was most commonly seen in the left eye (58.7%). An intergroup comparison was performed using the Kruskal-Wallis test at the end of 3 and 5 months. The results suggest that the success rate was significantly higher in patients with a silicone stent (P=0.04) as compared with the other two groups, although no significant difference in failure rate was seen between patients on mitomycin C and conventional therapy (P=0.132 at Month 3 and P=0.481 at Month 5, Mann-Whitney U-test).

**Conclusion:**

Our study shows that silicone tube stenting had a better success rate compared with the other two groups, with no significant statistical difference between the use of mitomycin C and the conventional technique.

## Introduction

In the current era, persistent symptomatic nasolacrimal duct (NLD) block is a very common infection among all age groups, and is encountered in clinical practice by both ophthalmologists and otorhinolaryngologists alike. Otorhinolaryngologists have a more refined approach in terms of preservation of lacrimal pump function by preserving the orbicularis oculi muscle and cosmesis (1).

Dacrocystitis is classified into primary and secondary conditions. Linberg and McCormick coined the term primary acquired NLD obstruction, in which the condition is caused by inflammation or fibrosis without any precipitating factors (2). Bartley classified secondary NLD obstruction in which the obstruction is secondary to trauma, malignancy or surgical interventions such as total maxillectomy (3–5). Dacrocystorhinostomy (DCR) is a very common procedure performed over the last 10 decades, either by an external approach or through an endoscopic approach. The aim is to re-establish lacrimal drainage by probing or surgical communication between the lacrimal sac and the nasal cavity in which lacrimal flow is diverted into the nasal cavity, through an artificial opening made at the level of the lacrimal sac (6).The endonasal approach for dacrocystitis was first surgically addressed by Caldwell in 1893, by removing the portion of the inferior turbinate and following the NLD pathway to the lacrimal sac. However, this approach failed to gain popularity because of limited access to the anatomy of the NLD (7–9). In 1904, an Italian rhinologist, Adeo Toti, who is also referred to as the father of external DCR, pioneered the technique of external DCR among ophthalmologists, which many centers still practice today as the technique of external DCR for dacrocystitis (9,10).

The credit for performing the first successful endonasal DCR goes to McDonough and Merring in 1989, but it was actually Rice in 1988 who performed the first successful endonasal DCR on a cadaver (9-11).

Numerous modifications have been introduced, with the use of various antifibrotic agents and prosthesis to maintain lacrimal patency. Brown advocated the use of mitomycin C derived from Streptomyces caespitose, which has an antifibrotic action and prevents the closure of a surgically created neo-ostium. In 1993, Kurihashi advocated the use of a transcanalicular silicon stent to be kept for few weeks, to maintain the patency of the surgically created neo-ostium (11). The objective of this study was to analyze whether the use of silicon tubing or mitomycin C offers an added advantage over conventional endonasal DCR.

## Materials and Methods

This was a randomized controlled trial of 150 patients who had acquired primary NLD obstruction, conducted at Grant Government Medical College, Mumbai between February 2014 and February 2017. Institutional ethical clearance was obtained prior to initiating the study, and informed consent was obtained from all patients. The aim of the study was to compare outcomes with respect to success rates and complications of endonasal DCR with application of mitomycin C vs. endonasal DCR using silicon stenting and conventional endonasal DCR as a control. Patients were randomly divided into three groups of 50 patients each based on a colored chit allocation.

**Fig 1:**
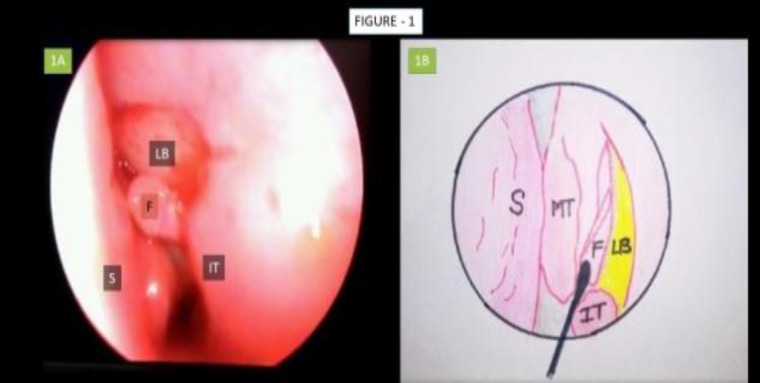
1A, Endoscopic image depicting elevated mucosal flap; 1B, Pictorial depiction of mucosal flap elevation S: Nasal Septum; MT: Middle Turbinate; F: Mucosal Flap; IT: Inferior Turbinate; LB: Lacrimal Bone

Inclusion criteria included 1) Adult patients (6 to 70 years) suffering from acquired NLD obstruction with or without mucopurulent discharge; 2) Delayed regurgitation with or without mucopurulent discharge from the opposite punctum on sac syringing examination. Exclusion criteria included 1) Patient suffering from other causes of epiphora such as eyelid malposition. entropion etc; 2) Sac syringing examination confirming common canalicular block; 3) Revision endonasal DCR; secondary NLD block due to NLD trauma, total maxillectomy etc. All 150 patients underwent endonasal DCR surgery under general anesthesia. The operative steps were as follows (Fig.1). 

**Fig 2:**
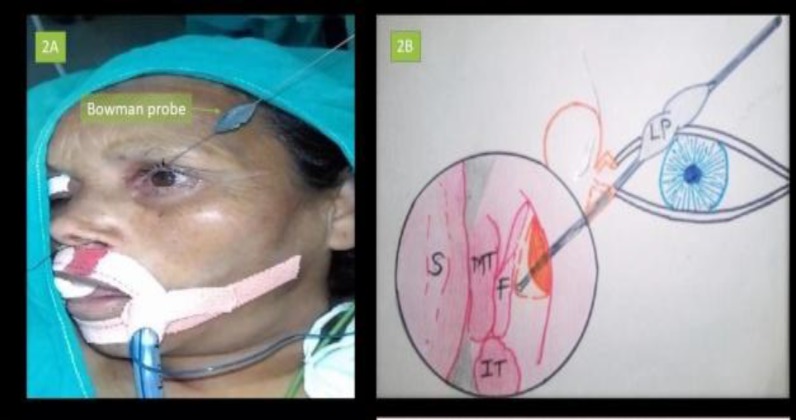
2A, Lacrimal probing with Bowman’s technique; 2B, Pictorial depiction of Bowman’s technique S: Nasal Septum; MT: Middle Turbinate; F: Mucosal Flap; IT: Inferior Turbinate; LP: Lacrimal Probe (Bowman

Initially decongestion of the middle turbinate and space anterior to the attachment of middle turbinate was performed using a rigid 30° endoscope for the procedure. The surgery was initiated by assessing the nasal septum, particularly for any significant deflection of the axilla of the middle turbinate (MT) which may be corrected. Out of 150 patients, septoplasty was performed in 10 patients and concha bullosa excision in 13 pts. Using a sickle knife, a rectangular incision was made anterior to the attachment of the MT., then the incised part of the lateral nasal wall was elevated using a Cat’s paw elevator (Fig 1). Next, a Kerrison’s punch was used to punch out the lacrimal bone for better exposure of the lacrimal sac, as confirmed by application of external pressure over the eyeball. Bowman’s technique was the performed using a lacrimal probe (Fig 2).

**Fig 3:**
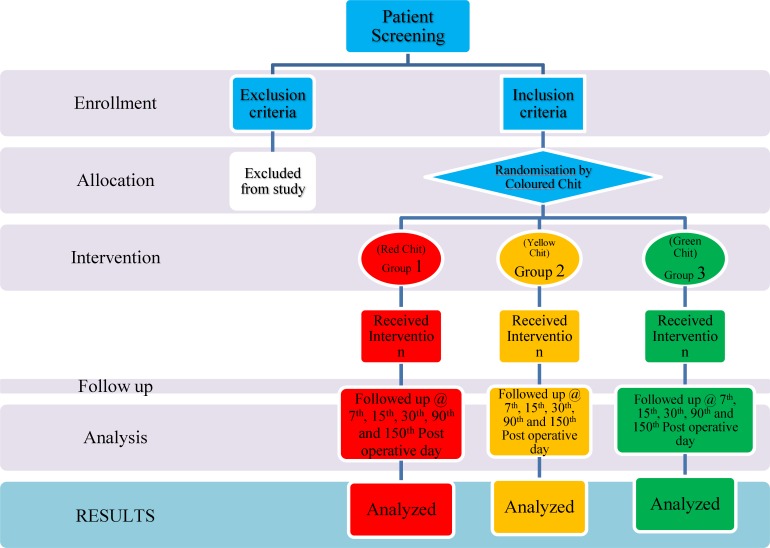
Randomisation Flow Chart

Finally, the lacrimal sac was incised using a No. 10 blade, and the flap was removed. At this point, the patients were divided into three groups (Fig.3). 

**Fig 4:**
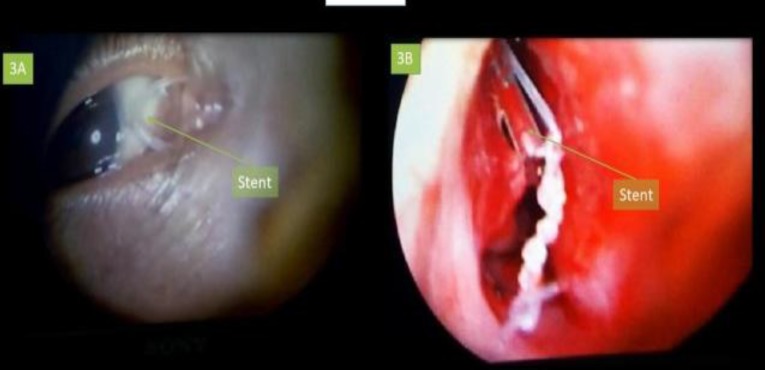
A, Silicon stent in lacrimal canaliculus; B, Endoscopic image of stent in nasolacrimal duct

In Group 1, patients with red-colored chits underwent endonasal DCR with application of mitomycin C at the stoma site; in Group 2, patients with orange-colored chits underwent endonasal DCR using silicon tubing to keep the stoma site patent for a period of 6 weeks (Fig.4 A,B); in Group 3, patients with green-colored chits underwent conventional endonasal DCR leaving the wide neo-ostium unchanged.

**Fig 5:**
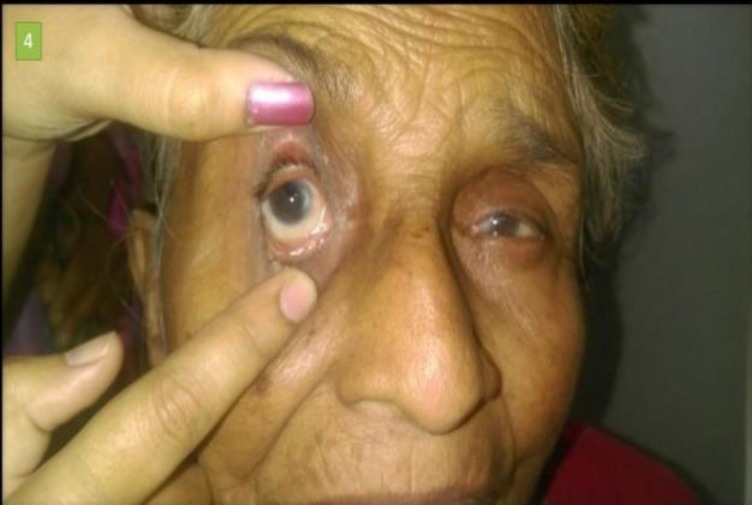
3 months’ postoperative image showing stent in situ

Following the procedure, all patients were put on oral antibiotics and anti-inflammatory drugs. After nasal pack removal, patients were given nasal decongestants and saline nasal douching. The patients were instructed to performed digital massaging on the external aspect of the lacrimal sac. All patients were followed up on Day 7, 15, 30, 90 and 150 after the procedure. In Group 1 and Group 2, sac syringing was performed at every follow-up visit, while in Group 3, sac syringing was performed once on the day of stent removal (Fig 5).

Surgical outcomes in terms of success and failure were evaluated both subjectively and objectively. In the subjective evaluation, the degree of epiphora relief was graded based on the scale developed by Munk et al. (1990) (12). According to this scale, epiphora is evaluated by the patient as follows: Grade 0 – no epiphora; Grade 1 – occasional epiphora requiring drying or dabbing less than twice a day; Grade 2 –epiphora requiring drying 2–4 times a day; Grade 3 – epiphora requiring drying 5–10 times a day; Grade 4 –epiphora requiring drying>10 times a day; Grade 5 – constant tear flow. Patients classified as Grade 0 were considered a successful outcome.

Objective assessment was achieved by performing sac syringing with simultaneous nasal endoscopic examination. Failed cases were diagnosed on clinical grounds of persistent epiphora and sac syringing showing regurgitation through the punctum during the postoperative follow-up period.

## Results

Following the study, all required data were compiled. The objective assessment was performed by means of sac syringing and endoscopic evaluation. The surgery was considered successful in the case of relief of epiphora and endoscopic confirmation of patency of stoma with sac syringing and irrigation. Out of 150 patients, 98 (65.3%) were female and 52 (34.7%) were male. The maximum incidence was found in the fourth decade (28.7%), followed by the fifth decade (23.3%). Out of 150 cases, 17 (11.3%) had bilateral involvement, and 88 (58.7%) had left and 17 (11.3%) had right eye involvement (Table.1).

**Table 1:** Patient demographics

**Factor**	**Frequency**	**Percentage**
Age (years)		
10–20	8	5.3%
21–30	20	13.3%
31–40	43	28.7%
41–50	35	23.3%
51–60	32	21.3%
61–70	12	8.0%
Gender		
Female	98	65.3%
Male	52	34.7%
Laterality		
Left	88	58.7%
Right	45	30.0%
Bilateral	17	11.3%
Symptoms		
Epiphora	150	100.0%
Nasal obstruction	15	10.0%
Rhinitis	15	10.0%
Sinusitis	20	13.3%
Swelling over medial Canthus	25	16.7%
Associated nasal pathology		
Deviated nasal septum	33	22.0%
Concha bullosa	13	8.7%
Agger nasi cells	10	6.7%
Accessory middle turbinate	5	3.3%

At the end of 3 months, an intergroup comparison was performed using the Kruskal-Wallis test. Results suggested that the success rate was significantly higher in patients with a silicone stent (P=0.04) as compared with the other two groups, although no significant difference in the failure rate was seen between patients treated with mitomycin C and conventional (P=0.132, Mann-Whitney U-Test).

After 5 months, an intergroup comparison was performed using the Kruskal-Wallis test (Table.2). Results suggested that the success rate was significantly higher in patients with a silicone stent (P=0.04) as compared with the other two groups, although no significant difference was seen in the failure rate between patients treated with mitomycin C and conventional therapy (P=0.481, Mann-Whitney U-Test). At the end of 5 months, 89% of patients were relieved of epiphora (Table.3). During the intraoperative, immediate postoperative period and on each follow-up visit, the patients were assessed for any surgical compilations. Five patients had immediate postoperative orbital emphysema which subsided within 48 hours with local treatment. Four patients in Group 3, three patients in Group 1 and two patients in Group 2 showed synechiae formation, which had cleared by the subsequent visit. Granulation formation in a stoma site was seen most commonly in Group 2 (six patients) followed by Group 3 (four patients) and Group 2 (two patients); granulation subsequently cleared on the next follow-up visit. The complication rate with mitomycin C was 14%, compared with 18% and 20% for silicon tubing stent and conventional endonasal DCR, respectively (Table 4).

**Table 2:** Postoperative patency using sac syringing under endoscopic vision

**Post-op follow up**	**Number of patients in follow up**	** Patients with patent** **stoma**	**Percentage (100%)**
Patients undergoing DCR using Mitomycin C			
Day 7	50	50	100.0%
Day 15	50	50	100.0%
Day 30	50	46	88.9%
3 months	45	40	88.7%
5 months	30	26	86.7%
Patients undergoing endonasal DC R using silicon stent			
Day 7	50	50	100.0%
Day 15	50	50	100.0%
Day 30	50	47	94.0%
3 months	50	45	90.0%
5 months	45	43	95.6%
Patients undergoing conventional endonasal DCR dacrocystorhinostomy			
Day 7	50	50	100.0%
Day 15	50	47	94.0%
Day 30	50	46	92.1%
3 months	49	43	87.8%
5 months	38	32	84.2%
			

**Table 3:** Subjective improvement in epiphora

**Munk Grading at 5 months**	**Type**			**Total** **(n=118)** **100%**
	**DCR + MMC n=30**	**DCR+ STENT** **n=50**	**Conventional n=38**	
Grade 0	26 (86.7%)	47 (94.0%)	32 (84.2%)	105 (89.0%)
Grade 1	0	0	0	0
Grade 2	0	0	0	0
Grade 3	0	0	0	0
Grade 4	04 (13.3%)	03 (6.1%)	6 (15.8%)	13 (11.0%)
Grade 5	0	0	0	0


**Table 4:** Complications

**Groups**	**Complications**			**Total no. patients**
	**Granulations**	**Synechiae**	**Orbital** **emphysema**	
Group I	2 (4.0%)	3 (6.0%)	2 (4.0%)	7 (14.0%)
Group II	6 (12.0%)	2 (4.0%)	1 (2.0%)	9 (18.0%)
Group III	4 (8.0%)	4 (8.0%)	2 (4.0%)	10 (20.0%)
Total	12 (24.0%)	9 (18.0%)	5 (10.0%)	26 (17.3%)

## Discussion

Endonasal DCR is accepted as a highly successful procedure in dealing with NLD obstruction. The current trends for the use of mitomycin C and stenting by silicon tubing are still being evaluated in comparison with conventional endonasal DCR. The choice of surgery generally rests at the hands of the surgeon, with many surgeons biased towards one particular method for performing DCR based on their practice and experience. No guidelines exist for selecting the optimal method for endonasal DCR surgery. Thus, uncertainty remains in terms of the best technique to be utilized to maintain patency or to prevent restenosis of the neo-ostium. There is also a lack of randomized controlled trials reported in the literature to compare the different methods of endonasal DCR.The aim of this study was to compare outcomes with respect to success rates and complications of endonasal DCR with administration of mitomycin C vs. endonasal DCR using silicon stenting and conventional endonasal DCR as a control. We divided the patients into three groups of 50 patients each based on a colored chit allocation (Fig. 3). Success was measured in terms of relief from epiphora and patency of the stoma as confirmed by sac syringing and endoscopic examination In this study, females (98) outnumbered males (52), with a male to female ratio of 1:1.9 (Table.1). Our study was comparable with other studies carried out by Zilelioglu et al. in Turkey who reported a male to female ratio 1:2 and Mudhol et al. in Indian who also reported a male to female ratio of 1:3 (13). It can be postulated that the frequent use of cosmetics such as kajal and exposure to smoke in the kitchen in a predominantly rural Indian setting increases the chances of dacrocystitis and stenosis among women.

The present study also showed a lower incidence of dacrocystitis in the elderly and young age groups, with a peak incidence seen in the fourth decade followed by fifth decade (Table.1). Forty-three patients out of 150 belonged to the age group 31–40 years (28.67%), with the fifth decade being the next most affected age group. These data correlate well with the studies done by Yung and Sperkelson, and a possible explanation for the declining trend in both extreme groups may be the decreased amount of lacrimal secretions (14,15).

A consideration of the side of the eye involved is also made in this study. In our study, 88 (58.6%) patients had left eye involvement and 45 (30.0%) had right eye involvement, while in 17 patients (11.3%) both eyes were involved (Table.1). Vishwakarma et al. reported similar results, with 63.3% of patients affected in the left eye. A possible explanation for this is that the nasolacrimal duct and lacrimal sac forms a greater angle on right side than on the left side, increasing the chances of stasis and obstruction of NLD on the left side. Other plausible explanations may be that most people are right handed, hence the left hand is free to clean the eye or wipe away tears, increasing the chances of infection on the left side. Congenital and anatomical narrowing on the left side are other theories proposed (7).

Out of 150 patients, 33 (22%) had a deviated nasal septum, 13 (8.67%) had concha bullosa, 10 (6.67%) had Agger Nasi cells, and five patients had accessory MT. Although 33 patients had deviated nasal septum, it was severe enough to limit access to the lacrimal sac region during surgery in 10 patients only, and these patients underwent limited endoscopic septal correction simultaneously.

The complications of endonasal DCR were mostly in the form of granulation, followed by synechiae formation. Granulations were found more commonly in Group 2; possibly as a response to foreign material (silicon tube). Synechiae was seen more in Group 3 followed by Group 1, and was seen least commonly in Group 2. The rates of complications were similar to those reported in the study conducted by Önerci et al. (16). Granulations were found more commonly in Group 2, which may be as a response to foreign material (silicon tube). Synechiae was seen more commonly in Group 3 followed by Group 1, and was seen least commonly in Group 2.

At the end of the 90^th^ day of follow up, 144 patients (96%) returned for evaluation. The success rate at the end of Day 90, as calculated based on Munk et al. scale, was 88.89% for Group 2, 90% for Group 2 and 87.75% for Group 3. An intergroup comparison was performed using the Kruskal-Wallis test. Results suggest that the success rate was significantly higher in patients with silicone stenting as compared with the other two groups (P=0.04), although those patients treated with mitomycin C and conventional therapy showed no significant difference in failure rates (P=0.132, Mann-Whitney U-Test). At the end of five months, a total of 118 patients (78.67%) were followed up; 30 in Group 1, 45 in Group 2 and 38 in Group 3. The success rate for Group I was 86.67%, with four patients having epiphora after the Day 90 visit. Success rates were 95% and 84.2% for Group 2 and Group 3, respectively.

The intergroup comparison was performed using the Kruskal-Wallis test. Results suggested that the success rate was significantly higher in patients in Group 1 (P=0.04) compared with the two other groups. However, there was no significant difference in the failure rates between Group 1 and Group 3 (P=0.481, Mann-Whitney U-test).

The Munk et al. scale for subjective improvement of epiphora that showed a significantly large number of individuals presenting with Grade 4 epiphora in DCR in Group 1 and Group 3 when compared with DCR in the Group 2. The success rate of endonasal DCR using mitomycin C in Group 1 was 86.67%. A study by Park et al. reported favorable outcomes in 87.8% patients. Our results are comparable with the success rate of endoscopic DCR using mitomycin C as reported in literature, which varied from 77–95% (17–19). The success rate endonasal DCR with silicone stenting in Group 2 was 95%. The study Bambuli and Chamero had a success rate of 91.7%, Fayet et al. reported 86%, while Weidenbacher reported rates of 95% with silicon stenting (20–23). Thus, correlation of the above-mentioned data suggests the success rate of endonasal DCR with silicon stent ranges from 82–95%, which is comparable with our study (20–22). In Group 3, the success rate of conventional endonasal DCR was 84.21%. The success rate of conventional endonasal DCR in the study conducted by Yang was 90.2%, while Tsirbas and Wormald reported a rate of 89% and Ambani et al. reported 88% (16,24). The success rates for conventional endonasal DCR varies from 88–90%, which is slightly higher than our study,at 84.2%(23–25).Thus, according to our study,Group 2 had marginally better outcomes in comparison with the other two groups, and the success rates were similar between Group 1 and Group 3.

## Conclusions

The study establishes endoscopic DCR as a relatively safe and effective procedure, which avoids a facial scar and allows for simultaneous treatment of nasal pathologies. A regular follow up is imperative to obtain a long-term result. The application of mitomycin C has a lower complication rate compared with silicon tubing and the conventional technique, but the surgical outcome is better with silicon tubing. On the other hand, silicon tubing has poor patient compliance and comfort and is expensive when compared with mitomycin C and conventional treatment. However, the role of an antimitotic such as mitomycin C in preventing fibrosis of neo-ostium in endonasal DCR needs further evaluation as we found no significant difference when compared with conventional endonasal DCR.
